# Time to Functional Outcome Optimization After Musculoskeletal Tumor Resection

**DOI:** 10.7759/cureus.27317

**Published:** 2022-07-26

**Authors:** Babe Westlake, Olivia Pipitone, Nicholas S Tedesco

**Affiliations:** 1 Orthopedic Surgery, Good Samaritan Regional Medical Center, Corvallis, USA; 2 Graduate Medical Education - Biostatistics, Good Samaritan Regional Medical Center, Corvallis, USA; 3 Orthopedic Oncology, Good Samaritan Regional Medical Center, Corvallis, USA

**Keywords:** patient outcome score, musculoskeletal tumor resection, orthopedic oncology, musculoskeletal tumor, musculoskeletal tumor society rating scale

## Abstract

Background

There is ample literature describing surgical outcomes after oncologic musculoskeletal tumor surgery, however, there is limited understanding of the time to optimization of functional outcome scores after resection. The purpose of this study was to identify the time to functional outcome optimization of Musculoskeletal Tumor Society (MSTS) scores after surgery for bone and soft tissue tumors and to identify factors correlated with recovery.

Methods

We retrospectively reviewed 187 patients from April 2016 to May 2021 that had undergone surgical treatment for musculoskeletal tumors. We assessed MSTS scores to determine the time to optimization and evaluated patient-specific and surgical factors for any influence on post-operative recovery.

Results

The majority of patients (92%) achieved their optimized score in one year or less. Eighty-two percent achieved the maximum MSTS score of 30. Osseous tumors, malignancy, adjuvant treatment with radiation and/or chemotherapy, deep location for soft tissue tumors, and bony work required for soft tissue tumors all significantly impacted time to MSTS score optimization.

Conclusion

The majority of patients with musculoskeletal tumors undergoing surgery can be expected to improve up to one year postoperatively. Those with bone tumors, malignant tumors, treatment with radiation and/or chemotherapy, deep soft tissue tumors, and bony work for soft tissue tumors can expect to have a longer recovery time and are at higher risk for not achieving premorbid functionality.

## Introduction

Musculoskeletal neoplasms can be benign or malignant. Benign soft tissue tumors have been reported to be more than 300 times more common than their malignant counterparts [1]. Sarcomas are a rare subset of malignant musculoskeletal tumors, with most subtypes having an incidence of one to five per 1,000,000 [2]. Metastatic carcinomas in the bone and soft tissues of the extremities and trunk are far more common. In patients with metastatic breast and prostate cancer alone, the incidence of bone metastasis ranges from 60-70% [3]. The surgical treatment of primary malignant musculoskeletal neoplasms often involves either amputation or local resection when anatomically possible. Surgical treatment of metastatic bone disease most often involves stabilization and antiresorptive medical therapy, however, some evidence exists for resection in solitary or oligometastatic disease depending on the tumor type and timing of metastases in the disease process [4].

In 1993, Enneking et al. published their work on the development of the Musculoskeletal Tumor Society Functional Evaluation (MSTS) score [5]. The MSTS score is a validated tool for physicians to objectively evaluate patients' functional status after treatment for benign and malignant bone and soft tissue tumors. The MSTS score has been found to have good interobserver reliability and has been shown to correlate well with other functional outcome scores for endoprosthetic reconstruction [6]. Functional outcomes in metastatic disease of bone have also been evaluated using the MSTS score [7-8]. Multiple studies have characterized expected functional outcome scores at various final follow-up times, however, it is unknown how long it takes for functional outcome scores to optimize and what factors impact functional outcomes [9-11]. This has implications regarding patient expectations, informed consent, determining appropriate follow-up schedules, and managing interventions for those not progressing as anticipated.

The purpose of this study was to determine the length of time it takes for patients’ MSTS scores to optimize after surgical treatment for benign and malignant bone and soft tissue tumors. We also evaluated multiple tumor and patient characteristics that could influence the time to optimization of MSTS functional outcome scores after surgical treatment for bone and soft tissue tumors. This work was previously presented as a poster at the Musculoskeletal Tumor Society Meeting on October 6-8, 2021.

## Materials and methods

After approval by the Samaritan Health Services Regional Institutional Review Board (approval number IRB20-013), a retrospective review of prospectively obtained data was performed on all patients with a bone or soft tissue tumor that were treated surgically by one surgeon from April 2016 through May 2021. Patients were excluded if they did not receive surgical treatment or did not have enough follow-up data to determine MSTS score optimization. If a patient had more than one surgical procedure in the study timeframe, only their first procedure was included. Patients’ MSTS scores were assessed at each of their postoperative visits, which were standardized to two weeks, six weeks, three months, six months, and 12 months for benign tumors, and two weeks, six weeks, three months, and every three months thereafter until mortality or two years postoperatively for malignant tumors. Malignancies were then additionally followed every six months for the third year postoperatively, and then yearly thereafter. Scores were considered optimized if they did not increase over two consecutive postoperative visits or reached the maximum MSTS score of 30.

We identified several potential factors that could influence postoperative recovery and MSTS score optimization, including patient age, sex, diabetes, anxiety or depression, tobacco use, BMI, tumor type (bone vs. soft tissue), tumor malignancy (malignant vs. benign), tumor location (upper extremity, lower extremity, trunk), adjuvant chemotherapy or radiation treatment, depth of tumor for soft tissue tumors in relation to investing muscular fascia, and type of surgery for bone tumors (resection alone vs. all other procedures).

First, Mann-Whitney U tests and Spearman rank correlations were used to determine whether patient characteristics (age, sex, BMI, diabetes, anxiety or depression, tobacco use at time of surgery) were significantly associated with days to MSTS score optimization. Then, linear regression models were used, predicting days to score optimization (which was log-transformed to uphold model assumptions) by each tumor/treatment characteristic (tumor type, malignancy, location, depth, chemotherapy or radiation treatment, and type of surgery) when adjusting for tobacco use, as that was the only patient characteristic significantly associated with days to MSTS score optimization. All analyses were performed in R version 3.6.1. (R Foundation for Statistical Computing, Vienna, Austria).

## Results

A total of 254 procedures were identified over the study period. Sixty had not completed enough follow-up to determine MSTS score optimization, and seven patients had more than one procedure, leaving 187 patients for analysis. The characteristics of the study population are listed in Table 1. The average age at the time of surgery was 51 years and 58% of the population was female.

**Table 1:** Patient and tumor characteristics (N=187) a This only includes bone tumors. The denominator in all percentages is 60. b Other complications included: nerve palsy (n=5), foot drop (n=1), arthrofibrosis (n=1), humeral shaft fracture around implant treated non-operatively (n=1), intra-op MCL rupture (n=1), nerve palsy and post-op infection requiring operative irrigation and debridement (n=1), DVT and post-op hematoma managed conservatively (n=1), nerve palsy, tibial stress fracture, and disease recurrence (n=1)

	Percentage (%)	Number
Average Age at Time of Surgery (SD)	50.6 (20.2)	
Min, Max	10, 87	
Sex		
Male	42%	79
Female	58%	108
Race/Ethnicity		
White	93%	173
Black	2%	3
Asian/Pacific Islander	2%	4
Hispanic/Latino	3%	6
Native American	1%	1
Average BMI (SD)	29.2 (7.0)	
Min, Max	15.3, 56.0	
Diabetes	13%	24
Anxiety or Depression	30%	56
Tobacco Use		
At Time of Surgery	7%	14
Former	19%	36
Never	73%	137
Preop Narcotics	20%	37
Benign vs Malignant Tumor		
Benign	70%	131
Malignant	30%	56
Bone vs Soft Tissue Tumor		
Bone	31%	58
Soft Tissue	69%	129
Type of Tumor		
Benign Soft Tissue	53%	99
Malignant Soft Tissue	16%	30
Benign Bone	17%	32
Malignant Bone	14%	26
Type of Surgery, for Bone Tumors Only^a^		
Resection Alone	62%	36
Resection With Fixation	12%	7
Resection With Reconstruction	9%	5
Curettage Resection	3%	2
Fixation Alone	12%	7
Amputation	2%	1
Depth of Tumor		
Superficial	26%	48
Deep	74%	139
Tumor Location		
Upper Extremity	26%	48
Upper Extremity Acral	6%	12
Lower Extremity	44%	83
Lower Extremity Acral	18%	34
Trunk	5%	10
Chemotherapy	12%	23
Radiation	11%	21
Complications		
Wound Dehiscence	9%	17
Infection	4%	8
Return to OR	3%	6
Other Complications^b^	6%	12
Any of the Above Complications	19%	35
Mortality	6%	11

**Figure 1:**
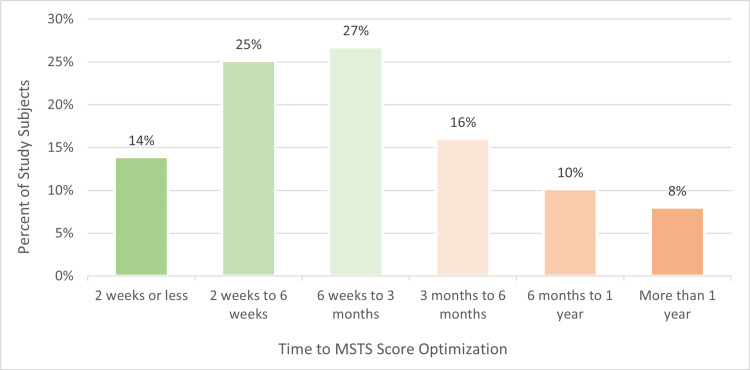
Time to MSTS score optimization (N=187) MSTS: Musculoskeletal Tumor Society

The average MSTS optimized score was 29.1 (97.0%) (SD=2.5, Min=14, Max=30) and 82% of the population (N=154) achieved the maximum possible score of 30 (100%) while 16% (N=29) had a maximum score of 20-29 (66.7-96.7%) and 2% (N=4) had a maximum score less than 20 (<66.7%). Figure 1 shows the time to optimization of MSTS scores for all tumor types. The majority of the population (66%) achieved their optimized score within three months, 26% achieved their optimized score within three months to one year, and 8% took more than one year to achieve their optimized score. The longest time to MSTS score optimization was 2.8 years. Of the 11 patients that died during follow-up, eight (73%) had reached the maximum MSTS score of 30 (100%). The remaining three patients reached optimized scores of 19, 24, and 27 (63.3, 80.0, and 90.0%, respectively).

Table 2 demonstrates the time to MSTS score optimization based on tumor characteristics. For the adjusted analyses, we explored whether age, sex, BMI, diabetes, anxiety or depression, or tobacco use were significantly associated with days to score optimization. Time to score optimization was shorter for tobacco users compared to patients not using tobacco at the time of surgery (p=0.04). No other patient characteristics were significantly associated with days to score optimization. Therefore, only tobacco use was adjusted for in the linear regression analyses.

**Table 2:** Days to score optimization a From linear regression, predicting days to score optimization, which was log-transformed to uphold model assumptions. Models were adjusted for patient diabetes status and tobacco use at the time of surgery. b Other procedures include resection with fixation (N=7), resection with reconstruction (N=5), fixation alone (N=7), curettage resection (N=2), and amputation (N=1).

	N	Average Days to Score Optimization (SD)	Median Days to Score Optimization (IQR)	P-value^a^
All Patients	187	112 (143)	50 (37-139)	-
Bone vs Soft Tissue				
Bone	58	164 (180)	93 (54-174)	<0.001
Soft Tissue	129	89 (116)	43 (20-92)	
Site				
Upper Extremity	60	95 (107)	45 (75-117)	Reference
Lower Extremity	117	123 (157)	64 (36-149)	0.24
Trunk	10	93 (163)	47 (42-50)	0.67
Benign vs Malignant				
Benign	131	88 (134)	43 (25 -86)	<0.001
Malignant	56	170 (147)	119 (76-212)	
Treatment				
Radiation and/or Chemo	29	151 (128)	89 (56-201)	0.02
No Radiation or Chemo	158	105 (145)	46 (35-111)	
Bone Tumors: Type of Procedure				
Resection Alone	36	141 (141)	92 (52-172)	0.3
All Other Procedures^b^	22	202 (229)	119 (76-255)	
Soft Tissue Tumors: Superficial vs Deep				
Superficial	48	76 (133)	41 (15-47)	0.03
Deep	81	97 (105)	48 (36-111)	
Soft Tissue Tumors: Bony Work				
Had Bony Work	10	166 (125)	133 (82-209)	0.004
No Bony Work	119	83 (113)	42 (198-85)	

Time to MSTS score optimization was significantly longer for patients with bone tumors and malignant tumors and for patients who underwent treatment with chemotherapy and/or radiation. The median time to optimization was 93 days for bone tumor patients, compared to 43 days for soft tissue tumors (p<0.001). Patients with malignant tumors had median days to optimization of 119, compared to 43 for benign tumors (p<0.001), and patients that underwent treatment with chemotherapy and/or radiation had a median time to optimization of 89 days, compared to 46 days for patients with no chemotherapy or radiation (p=0.01). In patients with soft tissue tumors, deep tumors had a significantly longer time to score optimization compared to superficial tumors (p=0.03, Median = 48 days vs 41 days), and bony work was also associated with a significantly longer time to score optimization (p=0.003, Median = 133 days vs 42 days).

## Discussion

To our knowledge, this study is the first to evaluate the time to optimization of functional outcome scores after bone and soft tissue resection. We found the mean time to optimization to be 112 days, which can be inferred to be between the three-month and six-month follow-up. Bone tumors, malignant tumors, adjuvant treatment with chemotherapy and/or radiation, deep soft tissue tumors, and bony work for soft tissue tumors significantly prolonged the time to functional optimization.

We found tobacco use at the time of surgery to be the only comorbidity or demographic variable in this study that influenced time to optimization. Surprisingly, tobacco use was associated with a shorter time to optimization of functional outcome scores. We found after post-hoc analysis that 71% of current tobacco users had benign soft tissue tumors, compared to only 51% of former users and non-tobacco users. Soft tissue tumors and benign tumors were associated with a shorter time to optimization, which may explain our unexpected trend for faster recovery in tobacco users. To our knowledge, there is no known association of tobacco use with benign soft tissue tumors. Further study may be warranted to corroborate or refute our finding that benign soft tissue tumors were more prevalent for tobacco users or to determine potential links between tobacco use and benign tumorigenesis. We may have also found this relationship to be significant because of the small patient population.

Less than 10% of our patient population took more than a year to reach their optimized score. It is possible that our patient population had relatively quick recovery times because the majority of the population was made up of patients with benign soft tissue tumors, which was found to be a significant predictor of faster time to optimization.

The time to optimize functional scores in our population is comparable to patients recovering from surgery in other orthopedic disciplines. Hoffart et al. demonstrated functional outcome scores to peak and then plateau between one and two years after total knee replacement [12]. In a study of elderly patients undergoing hip fracture surgery, Heikkinen and Jalovaara found most patients recovered the majority of activities of daily living by four months with less significant gains of function between four and 12 months [13]. Ly et al. found Sickness Impact Profile scores to significantly improve for up to two years after limb salvage from lower extremity trauma [14].

Previously, Oh et al. evaluated functional outcome scores after soft tissue sarcoma resection [15]. They found functional outcome scores to significantly improve until two years postoperatively and then plateau; contrary to our population in which the majority reached their optimized MSTS score within one year. It is possible the discrepancy is because our population included benign tumors, which we found to have a significantly faster time to recovery. Like our present study, Oh et al. also found bony work for soft tissue tumors to significantly impact the MSTS score. They found age to be a significant predictor of functional outcome scores in their population, but we did not [15].

Other studies have evaluated the final MSTS score over a given follow-up period but have failed to identify or report the time to optimization of the MSTS score [9-11,16-17]. When counseling patients, it is important to both inform them of what their final functional status will be as well as how long the recovery process will take. Until now, there has been little evidence beyond anecdote to guide providers in counseling patients regarding the time to recovery from these types of surgeries.

There are several limitations to our study. First, our study is retrospective in nature and therefore at risk for selection bias, and there is a possibility of unmeasured confounding variables that could have influenced our observed results. We tried to mitigate the risk of confounding variables by performing an adjusted analysis and exploring a number of potential patient demographics and comorbidities that could impact time to functional outcome score optimization. Additionally, this study used data from a single surgeon, which will help mitigate potential differences in postoperative management or recovery protocols that could be present if multiple surgeons were included. Second, we included all bone and soft tissue tumors as well as benign and malignant tumors because of the rarity of these diseases. This leads to a very heterogeneous study population. It is possible there are outliers in our population that could skew the results and may not actually represent the time to optimization for a particular subset of patients with musculoskeletal tumors. To avoid this pitfall, future work in this area could be done as a multicenter study to increase the sample size available for analysis. Additionally, we report time to optimization of MSTS scores in days even though we were not assessing patients daily. This may cause an overestimation of time to optimization because patients may have reached their optimized scores prior to being seen for their next follow-up visit. Alternatively, we could have treated time to MSTS optimization as a categorical variable, representing the post-operative follow-up schedule (two weeks, six weeks, three months, etc). We decided against this because (1) patients were not always able to follow up at the suggested postoperative appointment timeframes so some were seen several weeks early or late compared to their intended follow-up timeframe, and (2) that would have necessitated the use of multinomial logistic regression, and there would have been relatively small sample sizes in some of our outcome groups, which would reduce the power of our analysis compared to the linear regression analysis we ultimately performed. Finally, the use of the MSTS instead of other tools for measuring functional outcomes, such as the Toronto Extremity Salvage Score (TESS), may be seen as a limitation [18]. The MSTS has been criticized for not evaluating a patient’s perception of health or breadth of function and for having ceiling effects [19]. We chose to use the MSTS for several reasons: (1) it is a disease-specific measure of functional status, (2) it can be used for patients that have undergone limb salvage surgery or amputation, and (3) it evaluates outcomes other than physical impairment or disability, including pain and emotional acceptance [5]. We did not use the TESS because it was developed specifically for patients undergoing limb salvage surgery, and it strictly evaluates measures of physical disability [18].

## Conclusions

In conclusion, the majority of patients undergoing surgery for bone and soft tissue tumors can be counseled that they will likely reach maximum recovery before or at one year. Patients with bone tumors, malignant tumors, soft tissue tumors requiring bony work/reconstructions, deep soft tissue tumors, and treatment with chemotherapy and/or radiation can all be reasonably counseled that they can expect a longer recovery time. This study adds valuable information for clinicians to counsel patients with musculoskeletal tumors, especially of the benign type as malignant tumors garner more attention in the literature.
